# Three-Dimensional Volumetric Evaluation of the Sella Turcica and Sphenoid Sinus in Individuals with Unilateral Palatally Impacted Maxillary Canines Using CBCT

**DOI:** 10.3390/diagnostics16071098

**Published:** 2026-04-05

**Authors:** Manolya İlhanlı, Şerife Tuğçe Hasoğlan, Seçil Aksoy, Kaan Orhan

**Affiliations:** 1 Multidisciplinary Clinic, Faculty of Dentistry, Ondokuz Mayıs University, 55270 Samsun, Turkey; 2 Department of Orthodontics, Faculty of Dentistry, Near East University, 99138 Mersin, Turkey; serifetugce.hasoglan@neu.edu.tr; 3 Department of Dentomaxillofacial Radiology, Faculty of Dentistry, Near East University, 99138 Mersin, Turkey; secil.aksoy@neu.edu.tr; 4 Department of Dentomaxillofacial Radiology, Faculty of Dentistry, Ankara University, 06500 Ankara, Turkey; knorhan@dentistry.ankara.edu.tr; 5 Medical Design Application and Research Center (MEDITAM), Ankara University, 06590 Ankara, Turkey; 6 Department of Oral Radiology, School and Hospital of Stomatology, Cheelo College of Medicine, Shandong University, Jinan 250012, China

**Keywords:** sella turcica, sphenoid sinus, impacted canine, CBCT, cranial base, volumetric analysis

## Abstract

**Background/Objectives**: The sella turcica and sphenoid sinus are anatomically adjacent structures within the cranial base and may reflect variations related to craniofacial development. However, evidence regarding their three-dimensional characteristics in individuals with impacted canines remains limited. This study aimed to evaluate the morphological, linear, and volumetric characteristics of the sella turcica and sphenoid sinus in individuals with unilateral palatally impacted maxillary canines using cone-beam computed tomography (CBCT). **Methods**: This study included CBCT scans of individuals with unilateral palatally impacted maxillary canines and a control group. Linear measurements and morphology of the sella turcica were assessed. Sella turcica volume was calculated using both a geometric formula and voxel-based three-dimensional segmentation. Sphenoid sinus pneumatization patterns and volumes were also evaluated. Agreement between volumetric measurement methods was assessed using Bland–Altman analysis, and correlations between sella turcica and sphenoid sinus volumes were also analyzed. **Results**: Most morphological and volumetric parameters of the sella turcica and sphenoid sinus were comparable between groups. Among the linear measurements, only sella width was significantly greater in the control group, whereas other dimensions showed no significant differences. The distribution of sella turcica morphology and sphenoid sinus pneumatization patterns was similar in both groups. No significant differences were observed in sella turcica or sphenoid sinus volumes. Bland–Altman analysis demonstrated good agreement between geometric and voxel-based volumetric measurements. In addition, no significant correlation was identified between sella turcica and sphenoid sinus volumes. **Conclusions**: Unilateral palatally impacted maxillary canines were not associated with substantial morphological or volumetric alterations of the sella turcica or sphenoid sinus. These findings suggest that variations in these cranial base structures have limited value as indicators of unilateral palatal canine impaction.

## 1. Introduction

The sella turcica is a key anatomical structure located on the intracranial surface of the sphenoid bone along the midline of the cranial base. It houses the hypophyseal fossa, which contains the pituitary gland and plays an essential role in neuroendocrine regulation [1]. Adjacent to this structure lies the sphenoid sinus, a paired pneumatic cavity within the sphenoid bone that is usually divided by a septum. Beyond their anatomical proximity, the sella turcica and sphenoid sinus are also related from a developmental perspective, as both arise within the sphenoid complex during cranial base formation [2]. The basisphenoid contributes to the posterior sphenoid body, including the sellar region, while the sphenoid sinus later develops within this anatomical complex. Therefore, sphenoid sinus morphology may be associated with morphologic characteristics of the adjacent sellar and cranial base region. Recent studies have suggested a significant association between sphenoid sinus morphology and sella turcica morphometry [3,4].

Maxillary canines are the second most frequently impacted permanent teeth after third molars, and impaction of these teeth represents one of the most common eruption disturbances in the permanent dentition. Unilateral impaction occurs two to three times more frequently than bilateral impaction [5]. Palatal impactions are reported to occur more frequently than buccal impactions [6]. Although several etiological factors have been proposed, including genetic predisposition and disturbances in the eruption pathway, the underlying mechanisms responsible for palatal canine impaction remain incompletely understood.

From a developmental perspective, the sellar region occupies an important interface within craniofacial morphogenesis. A neuro-osteological study has suggested that the axial skeleton up to the sella turcica is closely related to notochordal development, whereas the craniofacial skeleton anterior to the sella develops from prechordal cartilage, invading mesoderm, and neural crest cells [7]. Likewise, tooth development is initiated within the oral epithelium and proceeds through reciprocal interactions with neural crest-derived ectomesenchyme [8]. During early embryogenesis, neural crest cells migrate into and contribute to the facial prominences, including the frontonasal and maxillary processes, which are directly involved in the formation of the upper jaw [7]. The dental epithelium appears early in these regions and subsequently gives rise to the dental lamina and tooth primordia [8]. Within this developmental context, variation in the sellar–sphenoid complex may be associated with craniofacial features relevant to eruption disturbances. Several studies have therefore suggested that variations in the morphology of the sella turcica may serve as indicators of developmental anomalies such as hypodontia, hyperdontia, tooth impaction, and transposition [9,10].

Among these anomalies, the relationship between palatally impacted canines and sella turcica morphology has attracted considerable interest. Several studies have reported a higher frequency of sella turcica bridging in individuals with impacted canines [11,12]. However, the available evidence remains inconsistent, as some studies identified a significant association, whereas others did not confirm this finding [13,14].

Most previous studies evaluating the relationship between impacted canines and cranial base structures have relied on two-dimensional imaging techniques, such as lateral cephalograms and panoramic radiographs [11,12,13,15]. However, these methods present inherent limitations due to the superimposition of anatomical structures and do not allow accurate three-dimensional evaluation of craniofacial anatomy. In contrast, cone-beam computed tomography (CBCT) provides high-resolution three-dimensional imaging with relatively low radiation exposure and has become an important tool for the assessment of craniofacial structures, including impacted teeth and paranasal sinuses [1,16]. Despite the growing use of CBCT in craniofacial imaging, studies simultaneously evaluating the morphological characteristics of the sella turcica and the sphenoid sinus remain limited [3,4,17]. Furthermore, only one study has evaluated the sella turcica volumetrically [18], and to the best of our knowledge, no study has comprehensively evaluated the three-dimensional volumetric relationship between the sella turcica and the sphenoid sinus, particularly in individuals with impacted canines. Previous CBCT-based studies exploring the association between impacted canines and the sella turcica have mainly focused on morphological characteristics or linear measurements rather than volumetric parameters [14,19]. Therefore, the present study aimed to comparatively evaluate the morphological and volumetric characteristics of the sella turcica and the sphenoid sinus using CBCT images in individuals with unilateral palatally impacted maxillary canines and in a control group without impacted teeth.

## 2. Materials and Methods

### 2.1. Ethical Approval and Study Design

This retrospective cross-sectional study was conducted in accordance with the principles of the Declaration of Helsinki and received ethical approval from the Near East University Health Sciences Ethics Committee (Approval No: YDU/2025/137-2011). All CBCT datasets were fully anonymized prior to analysis to ensure patient confidentiality.

### 2.2. CBCT Acquisition Parameters

CBCT images were obtained using two CBCT systems: a NewTom 3G unit (Quantitative Radiology s.r.l., Verona, Italy) and a Sirona Orthophos SL 3D unit (Dentsply Sirona, Bensheim, Germany). The NewTom 3G system operated at 120 kVp and 3–5 mA with a scan duration of 36 s and a voxel size of 0.3 mm. The field of view (FOV) ranged between 9 and 12 inches. The Orthophos SL 3D unit operated at 60–90 kVp and 3–16 mA with a 14 s exposure time and a voxel size of 0.08 mm, using a field of view of 110 × 100 mm.

During image acquisition, patients were positioned according to the manufacturer’s standard protocol. For the NewTom 3G system, scans were obtained in the supine position, whereas the Orthophos SL 3D system acquired images with the patient in an upright position. Laser positioning guides and bite stabilization devices were used when appropriate to standardize head alignment and occlusion. All patients were instructed to remain motionless during scanning.

All CBCT datasets were exported in DICOM (Digital Imaging and Communications in Medicine) format for subsequent three-dimensional reconstruction and analysis.

### 2.3. CBCT Dataset and Study Population

CBCT scans obtained between 2010 and 2025 at the Department of Dentomaxillofacial Radiology, Faculty of Dentistry, Near East University, were retrospectively analyzed. Individuals aged 12 years or older who had undergone CBCT imaging for various dental indications were considered eligible for inclusion.

Images were excluded in cases of maxillofacial trauma, craniofacial anomalies or syndromes (including cleft lip and/or palate), odontogenic lesions in the region of interest, obstructive sleep apnea, previous orthodontic or orthognathic treatment, systemic conditions affecting craniofacial development, absence of relevant anatomical structures within the field of view, severe motion artifacts, or poor image quality.

In the control group, scans showing any impacted teeth other than third molars were excluded. To minimize potential confounding, individuals with developmental dental anomalies that might affect sella turcica morphology, including hypodontia, hyperdontia, tooth transposition, or other eruption disturbances, were also excluded. The unilateral impacted canine (UIC) group consisted exclusively of individuals with a unilateral palatally impacted maxillary canine and no additional impacted teeth (excluding third molars).

### 2.4. Sample Size Calculation

An a priori sample size analysis was performed using G*Power software (version 3.1.9.7, Heinrich-Heine-Universität Düsseldorf, Düsseldorf, Germany, http://www.gpower.hhu.de/, accessed on 12 October 2025). Based on a two-tailed independent sample *t*-test, assuming a moderate-to-large effect size of 0.70, an alpha level of 0.05, a statistical power of 80%, and equal group allocation, the minimum required sample size was calculated as 34 participants per group. To account for possible exclusions, 70 patients were ultimately included in the study.

### 2.5. Study Groups

Two study groups were established following archive screening:

- Control group (*n* = 35): Individuals without impacted teeth (excluding third molars).
- UIC group (*n* = 35): Individuals presenting with a unilateral palatally impacted maxillary canine (excluding third molars).

The age and sex distributions were comparable between the groups. Each group consisted of 29 females and 6 males.

### 2.6. Image Processing and 3D Segmentation

All CBCT datasets were converted to the Digital Imaging and Communications in Medicine (DICOM) format and imported into 3D Slicer software (version 5.8.1; http://www.slicer.org, Boston, MA, USA), an open-source platform for medical image analysis and visualization. 

Segmentation of the sella turcica and sphenoid sinus was performed using the Segment Editor module following a semi-automated workflow. Initially, intensity-based thresholding was used to generate a preliminary binary mask for low-density and air-filled regions. This was followed by manual refinement using the Scissors and mask-editing tools, with segmentation boundaries adjusted on a slice-by-slice basis in the axial, sagittal, and coronal planes to remove artifacts and exclude anatomically irrelevant regions.

Segmentation criteria were anatomically defined. The sella turcica was segmented within its predefined osseous boundaries without extension into adjacent cranial base structures, whereas the sphenoid sinus segmentation included the pneumatized sinus cavity while excluding surrounding cortical bone and adjacent non-sinus structures.

**Figure 1:**
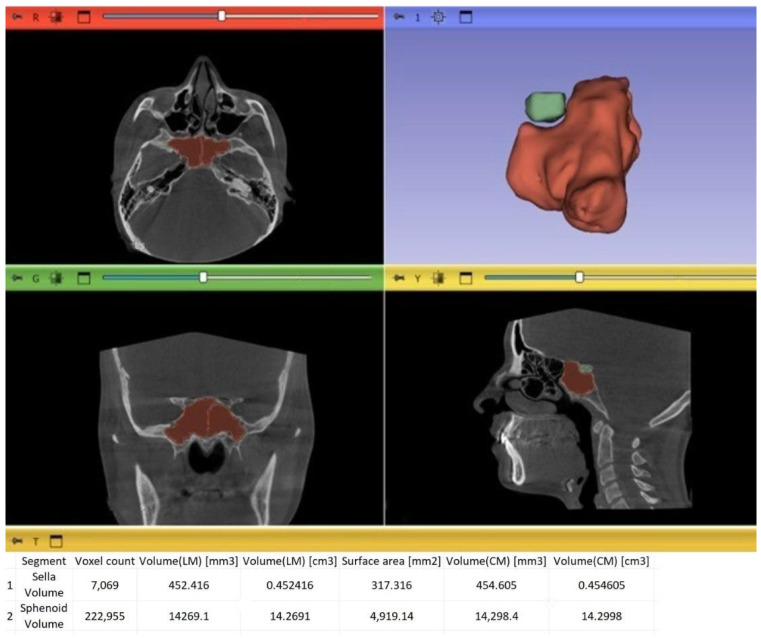
Visualization of the sphenoid sinus (red) and sella turcica (green) on sagittal and coronal CBCT slices, along with 3D segmentation.

Segmentation accuracy was ensured through combined evaluation of two-dimensional slice views and three-dimensional surface reconstructions. Region-growing and mask-editing tools were used as needed to refine segmentation boundaries and remove artifacts. Final segmentations were visually verified using three-dimensional surface reconstructions (Figure 1). Volumetric measurements were obtained using the “Segment Statistics” module based on voxel summation and were reported in cubic millimeters (mm^3^).

All measurements were performed by a single calibrated examiner (Ş.T.H.), who was blinded to patient information and clinical records during image evaluation. To evaluate intra-observer reliability, 50% of the scans were randomly selected and reanalyzed after a one-month interval and intraclass correlation coefficients (ICC) were calculated. Particular attention was paid to maintaining anatomical accuracy during segmentation, and the boundaries of each structure were carefully verified in axial, sagittal, and coronal planes. All image evaluations were performed under standardized viewing conditions using the same workstation, monitor, and software settings to ensure measurement consistency.

### 2.7. Linear Measurements of the Sella Turcica

**Figure 2:**
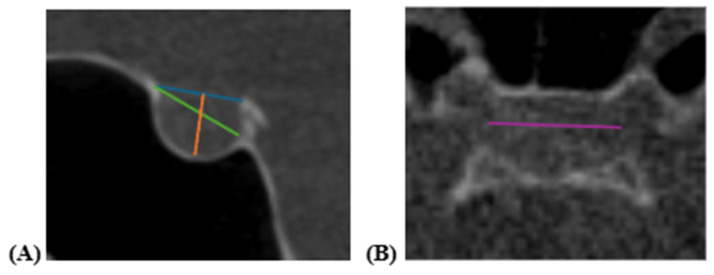
Linear measurements of the sella turcica: (**A**) midsagittal view illustrating length (blue), depth (orange), and anteroposterior diameter (green); (**B**) axial view illustrating width (purple).

Head orientation was standardized using multiplanar reconstruction (axial, sagittal, and coronal planes) by aligning the Frankfort Horizontal plane parallel to the floor. Linear measurements were obtained from midsagittal and axial CBCT sections (Figure 2):

- Length: Distance between the tuberculum sellae and dorsum sellae (Figure 2A);
- Depth (height): Perpendicular distance from the line connecting the tuberculum sellae and dorsum sellae to the deepest point of the sella floor (Figure 2A);
- Anteroposterior diameter: Distance from the most superior point of the tuberculum sellae to the deepest point of the posterior wall of the sellar fossa (Figure 2A);
- Width: Maximum lateral distance between the right and left lateral walls measured on the axial plane (Figure 2B).

All measurements were recorded in millimeters (mm).

### 2.8. Skeletal Classification

The sagittal skeletal pattern was additionally assessed using the ANB angle measured on CBCT-derived lateral cephalometric images. The ANB angle was calculated as the difference between the SNA and SNB angles. Based on ANB values, subjects were classified into skeletal Class I, II, or III.

### 2.9. Volumetric Measurements

#### 2.9.1. Sella Turcica Volume

Sella turcica volume was calculated by three-dimensional segmentation of the region bounded anteriorly by the tuberculum sellae, posteriorly by the dorsum sellae, inferiorly by the sphenoid body, and superiorly by a line connecting the tuberculum sellae and dorsum sellae (Figure 1).

#### 2.9.2. Sphenoid Sinus Volume

Sphenoid sinus volume was determined by segmenting the radiolucent sinus cavity. The anatomical boundaries were defined as follows: anteriorly, the nasal cavity; posteriorly, the clivus and cranial base; superiorly, the sella turcica and pituitary region; inferiorly, the vomer and nasopharynx; and laterally, the lateral walls of the sphenoid sinus adjacent to the cavernous sinus (Figure 1).

#### 2.9.3. Geometric Volume Calculation

In addition to three-dimensional segmentation, sella turcica volume was also estimated using the geometric formula:

Volume = 0.5 × (length × width × depth)

### 2.10. Morphological Classification

**Figure 3:**
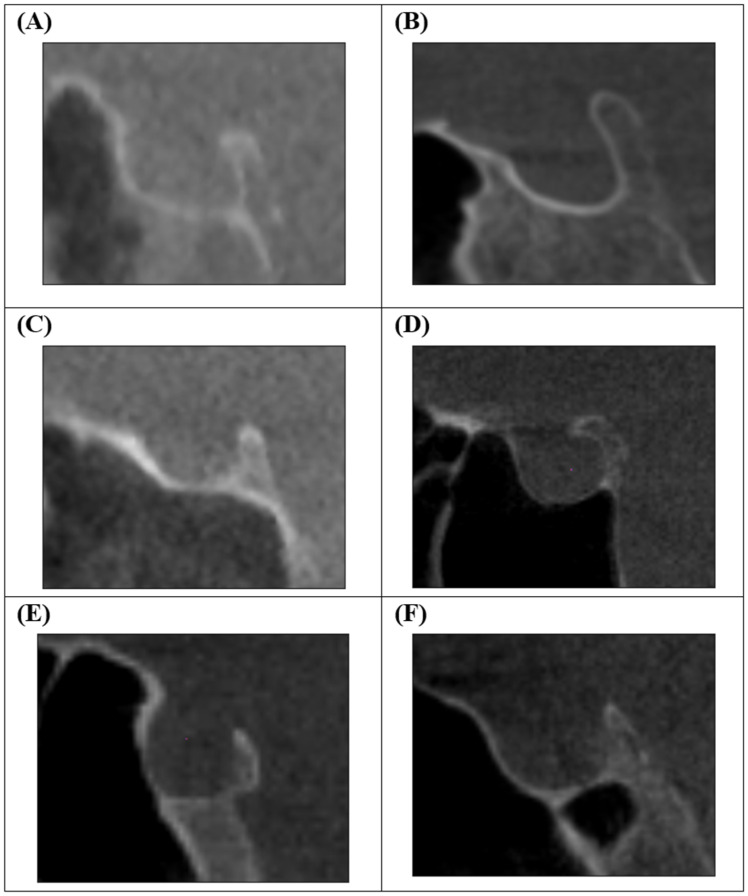
Morphological types of the sella turcica according to Axelsson et al. [20]: (**A**) normal, (**B**) oblique anterior wall, (**C**) double contour of the floor, (**D**) sella turcica bridge, (**E**) irregularity (notching) of the posterior part, (**F**) pyramidal shape of the dorsum sellae.

Sella turcica morphology was assessed according to the six-type classification described by Axelsson et al. [20] (Figure 3): normal morphology, oblique anterior wall, double contour of the floor, sella turcica bridging, irregularity of the posterior part, and pyramidal shape of the dorsum sellae.

**Figure 4:**
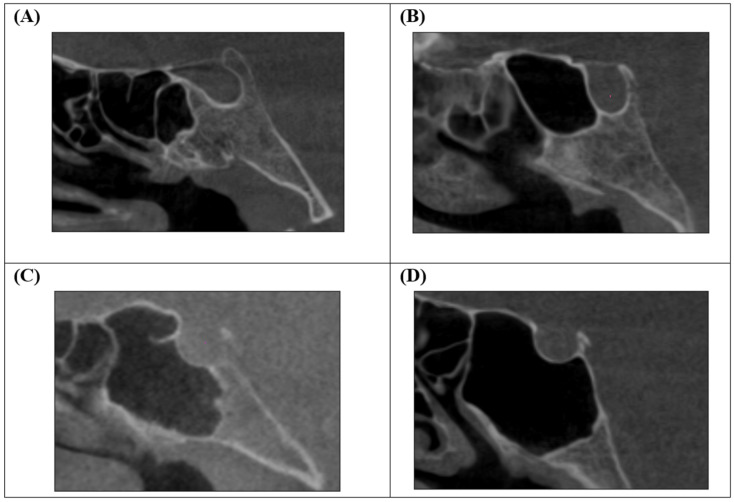
Sphenoid sinus pneumatization: (**A**) conchal, (**B**) presellar, (**C**) sellar, (**D**) postsellar.

The degree of sphenoid sinus pneumatization was evaluated according to the classification proposed by Şimşek et al. [21], which includes four types: conchal, presellar, sellar, and postsellar (Figure 4).

Statistical Analysis

All statistical analyses were performed using SPSS software (version 22; IBM Corp., Armonk, NY, USA). The normality of continuous variables was assessed using the Shapiro–Wilk test. Continuous variables were presented as mean ± standard deviation as appropriate.

Differences between the control and UIC groups were analyzed using the Mann–Whitney U test for non-normally distributed variables. Effect sizes for Mann–Whitney U comparisons were expressed as r values and interpreted according to Cohen’s conventions: small (0.1), medium (0.3), and large (0.5).

Categorical variables, including sex, sella turcica morphology, and sphenoid sinus pneumatization types, were compared using the Chi-square test, with Monte Carlo simulations applied when expected cell counts were low, and the strength of association was evaluated using Cramér’s V.

Comparisons between geometrically estimated and three-dimensional sella turcica volumes were performed using either paired *t*-tests or Wilcoxon signed-rank tests depending on the distribution of the data.

Pearson or Spearman correlation coefficients were used to assess associations calculated to evaluate relationships between sella turcica and sphenoid sinus volumes. A *p* value of <0.05 was considered statistically significant.

## 3. Results

### 3.1. Study Population

A total of 70 CBCT scans were included in the study, comprising 35 individuals in the control group and 35 individuals with unilateral palatally impacted maxillary canines. The demographic characteristics of the study population are summarized in Table 1. Participants in the UIC group ranged in age from 15 to 69 years, whereas those in the control group ranged from 15 to 67 years. No statistically significant differences were observed between the groups in terms of age or sex distribution (*p* > 0.05).

### 3.2. Linear Measurements of the Sella Turcica

The comparison of linear sella turcica measurements between the UIC and control groups is presented in Table 2. No statistically significant differences were observed between the groups in terms of anteroposterior diameter, length, or depth. Among all linear measurements, sella width showed the only statistically significant difference between groups (*p* = 0.006), with the control group exhibiting greater values.

Effect size analysis supported the group comparisons shown in Table 2. Among the linear measurements, sella width demonstrated the largest effect size (r = 0.33), indicating a small-to-moderate group difference, whereas the remaining parameters showed only small effect sizes (r = 0.06–0.17).

### 3.3. Morphological Characteristics

The distribution of sella turcica morphological types according to the Axelsson classification is shown in Table 3 [20]. The most frequently observed morphology in both groups was normal sella turcica morphology, followed by oblique anterior wall and posterior wall irregularities. No statistically significant differences were found between the control and UIC groups regarding sella turcica morphology. Overall, the distribution of morphological types did not differ significantly between groups (χ^2^(4) = 2.53, Monte Carlo *p* = 0.713, Cramér’s V = 0.19).

Similarly, the distribution of sphenoid sinus pneumatization types is presented in Table 4. The sellar type pneumatization was the most common pattern in both groups. No statistically significant differences were observed between the groups in terms of sphenoid sinus pneumatization patterns (χ^2^(3) = 4.581, *p* = 0.206, Cramér’s V = 0.256).

### 3.4. Skeletal Classification

No significant differences were observed between the control and UIC groups in terms of sagittal skeletal pattern. The mean ANB angle was 3.34 ± 2.10 in the control group and 2.94 ± 2.68 in the UIC group (*p* = 0.424). Likewise, skeletal class distribution was similar between the groups (*p* = 0.873). These findings suggest that sagittal skeletal morphology was comparable in the two groups.

### 3.5. Volumetric Measurements

The volumetric measurements of the sella turcica and sphenoid sinus are summarized in Table 5.

The mean three-dimensional sella turcica volume was lower in the UIC group than in the control group, but the difference did not reach statistical significance (*p* = 0.083). Likewise, the mean sphenoid sinus volume was lower in the UIC group; however, this difference was also not statistically significant.

Effect size estimates for volumetric measurements are presented in Table 5. The effect sizes were small for geometric sella volume (r = 0.14), three-dimensional sella turcica volume (r = 0.21), and sphenoid sinus volume (r = 0.11), indicating limited intergroup differences in volumetric parameters.

### 3.6. Inter-Device Variability Analysis

An additional subgroup analysis was performed to assess potential inter-device variability according to CBCT unit type. No statistically significant differences were found between the two CBCT units for geometric sella volume, three-dimensional sella turcica volume, or sphenoid sinus volume in either the control group or the UIC group. Similarly, no significant device-related differences were observed when all cases were analyzed together, suggesting that the use of two CBCT units did not introduce a detectable systematic bias in volumetric segmentation.

### 3.7. Correlation Between Sella Turcica and Sphenoid Sinus Volumes

**Figure 5:**
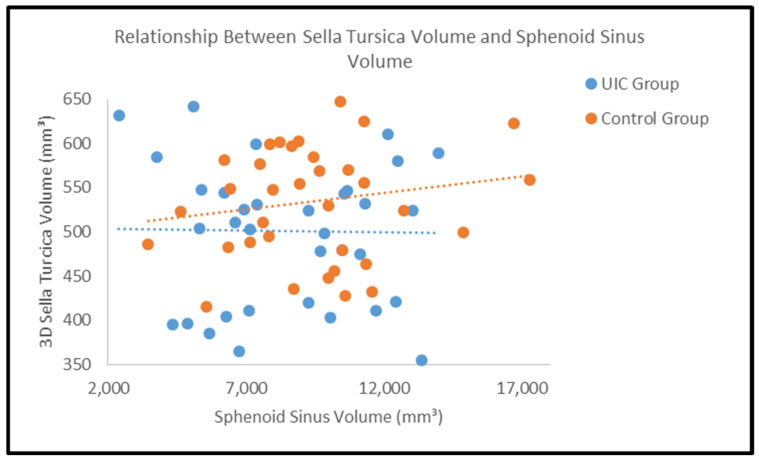
Scatter plot illustrating the relationship between three-dimensional sella turcica volume and sphenoid sinus volume in the UIC and control groups. Each point represents an individual subject. Dotted lines indicate the linear regression trends for each group.

Correlation analysis showed no significant association between sphenoid sinus volume and either geometric or three-dimensional sella turcica volume in either group (Table 6). In the control group, the correlation coefficient between sphenoid sinus volume and 3D sella volume was r = 0.104 (*p* = 0.554), whereas in the UIC group it was r = 0.037 (*p* = 0.833) (Figure 5).

### 3.8. Comparison of Volumetric Measurement Methods

Paired comparisons demonstrated no statistically significant differences between geometrically estimated and three-dimensional sella turcica volumes in either the control group (*p* = 0.071) or the UIC group (*p* = 0.099) (Table 7).

**Figure 6:**
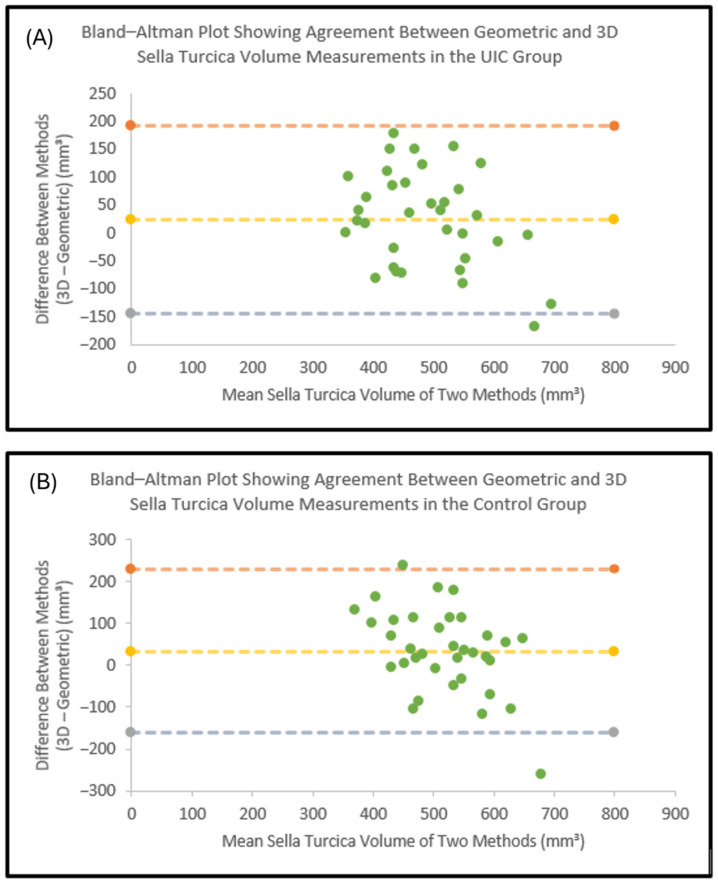
Bland–Altman plots showing the agreement between geometrically estimated sella turcica volumes and three-dimensional segmentation measurements. Green dots denote individual cases, the central yellow dashed line represents the mean difference (bias), and the upper and lower dashed lines represent the 95% limits of agreement. (**A**) UIC group; (**B**) Control group.

Bland–Altman analysis demonstrated acceptable agreement between geometrically estimated sella turcica volumes and three-dimensional volumetric measurements in both study groups. The majority of observations fell within the 95% limits of agreement, with no evident systematic bias across the measurement range (Figure 6).

### 3.9. Observer

Intraobserver reliability was assessed using ICC. The ICC values ranged from 0.819 to 0.998, indicating high reproducibility across all measurements. All ICC values were statistically significant (*p* < 0.0001), confirming the consistency and reliability of the measurement protocol (Table 8).

## 4. Discussion

The present CBCT-based study aimed to evaluate the relationship between unilateral palatally impacted maxillary canines and structural variations in the sella turcica and sphenoid sinus. Overall, the findings were predominantly negative: most morphological, linear, and volumetric parameters were comparable between the UIC and control groups, and no significant correlation was found between sella turcica and sphenoid sinus volumes. These results suggest that, despite their anatomical proximity, variations in these cranial base structures do not provide meaningful diagnostic utility for screening or predicting unilateral palatal canine impaction. The only significant difference observed was sella width, which was smaller in the UIC group; however, because this finding was isolated and not accompanied by broader morphological or volumetric differences, it should be interpreted cautiously rather than as a clinically useful marker.

### 4.1. Sella Turcica Morphology

The morphology of the sella turcica has long attracted attention in craniofacial and developmental studies, particularly because certain sellar variations have been linked to dental anomalies and altered eruption patterns [9]. In the present study, however, the overall distribution of sella turcica morphology was similar in the UIC and control groups. Normal morphology was the most common pattern in both groups, followed by irregularity of the dorsum sellae and pyramidal dorsum sellae, which is in agreement with previous descriptions in non-syndromic populations [20,22].

Earlier studies, particularly those based on lateral cephalograms, have reported associations between sella turcica bridging and canine impaction or other dental abnormalities [9,11,12,23]. In contrast, more recent investigations have yielded less consistent results [13,14]. The present findings are more consistent with these latter studies. Although sella turcica bridging was observed only in the UIC group, the overall distribution of morphological patterns did not differ significantly between groups. Taken together, these findings suggest that isolated sella turcica morphology, even when evaluated three-dimensionally, has limited value as a screening or predictive marker for unilateral palatally impacted maxillary canines.

This discrepancy between earlier and more recent studies may partly reflect differences in imaging modality, study design, or diagnostic criteria. Two-dimensional techniques are more susceptible to superimposition and projection errors and may overestimate certain morphologic variations [24]. By contrast, CBCT allows more accurate visualization of the sellar region in three dimensions. Therefore, the absence of a significant difference in the present CBCT-based study may reflect a more reliable three-dimensional assessment of sellar morphology, although limited sensitivity for subtle differences should also be considered.

In addition to morphology, linear dimensions of the sella turcica were assessed. Among these parameters, only sella width differed significantly between groups, being smaller in the UIC group, whereas length, depth, and anteroposterior diameter were comparable. Because this difference was limited to a single parameter and was not accompanied by corresponding differences in morphology or volume, its biological significance appears limited. Previous studies on sellar dimensions in impacted canine patients have reported inconsistent findings [14,23]. Baidas et al. [23] observed increased sellar dimensions in subjects with canine impaction, whereas Bavbek et al. [14] reported no significant association between impacted canines and sella turcica dimensions in a CBCT-based study. The present data do not support the presence of a consistent or clinically meaningful dimensional sellar phenotype associated with unilateral palatal canine impaction.

### 4.2. Sella Turcica Volume

A strength of the present study is the inclusion of both geometric and voxel-based three-dimensional volumetric assessment of the sella turcica. Geometric formulas are practical and easy to apply, but they assume an idealized shape and may not fully capture the anatomical complexity of the sellar cavity. Voxel-based segmentation, in contrast, permits direct measurement of the segmented structure and is therefore more anatomically representative.

Despite this more detailed three-dimensional approach, no significant intergroup differences were found in either geometric or voxel-based sella turcica volume. In addition, paired comparisons and Bland–Altman analysis demonstrated reasonable agreement between the two methods, although some individual variability was evident. This indicates that geometric estimation may be acceptable for gross approximation, but voxel-based segmentation remains preferable for detailed morphometric evaluation. More importantly, the lack of volumetric differences between groups suggests that sellar volume does not appear to be a useful indicator of unilateral palatal canine impaction. Thus, the present findings do not support sellar volumetric assessment as a clinically useful screening tool in this context.

### 4.3. Sphenoid Sinus Morphology and Volume

Because the sphenoid sinus and the sella turcica are anatomically adjacent and develop within the same cranial base region, it was reasonable to investigate whether sphenoid sinus morphology might vary in association with canine impaction [3,4]. In both groups, the postsellar type was the most frequent pneumatization pattern, in agreement with a previous imaging study [25]. Although the UIC group showed a slightly higher prevalence of postsellar pneumatization, the overall distribution of sphenoid sinus types did not differ significantly between groups.

At the same time, the sellar type was numerically more frequent in the control group than in the UIC group. Although this finding did not reach statistical significance, it may indicate a pattern worthy of further investigation. Because categorical subgroup frequencies were relatively small, particularly in infrequent categories, these results should be interpreted cautiously.

Similarly, sphenoid sinus volume did not differ significantly between groups. From a clinical perspective, this is an important negative finding. If sphenoid sinus morphology or volume were to have value as a screening or predictive marker, at least some consistent intergroup differences would be expected. The absence of such differences in the present CBCT-based study suggests that sphenoid morphology does not appear to have meaningful diagnostic utility for predicting unilateral palatal canine impaction. Although cranial base structures remain biologically relevant in developmental research, the present data do not support sphenoid sinus morphology or volume as reliable standalone markers in clinical screening.

### 4.4. Relationship Between Sella Turcica and Sphenoid Sinus Volumes

The close anatomical proximity of the sella turcica and sphenoid sinus within the sphenoid bone prompted further investigation of the potential relationship between their volumes. However, correlation analysis revealed only very weak and non-significant associations in both groups. Scatter plots also showed no evident linear trend. These findings suggest that the volumes of these adjacent structures vary largely independently, at least in the context of unilateral palatal canine impaction.

This result is also relevant from a developmental standpoint. Although these structures arise within the cranial base region, they are influenced by multiple genetic, embryological, and functional factors, and anatomical proximity alone does not necessarily imply coordinated volumetric development. Therefore, while developmental interactions cannot be excluded, the present findings do not support a meaningful combined volumetric marker involving the sella turcica and sphenoid sinus for the identification of unilateral palatally impacted maxillary canines.

Limitations

Several limitations should be considered when interpreting the findings of this study. First, the retrospective cross-sectional design may have introduced selection bias, since the CBCT scans were obtained for clinical indications rather than a standardized research protocol. Second, all image analyses were performed by a single examiner; although intraobserver reliability was high, interobserver reliability was not assessed. Third, the study focused specifically on unilateral palatally impacted maxillary canines, and therefore the findings may not be generalized to bilateral or buccally impacted canines.

Another limitation of this study is the sample size. The study was powered to detect moderate-to-large effects (d = 0.70) based on prior literature. However, the observed effect sizes for volumetric parameters were small (r = 0.11–0.21). These findings suggest that substantially larger samples would be required to reliably detect small effects.

In addition, the study was conducted at a single center and included a relatively homogeneous population. Because craniofacial and cranial base morphology may vary among populations, the generalizability of the present findings may be limited. Furthermore, although the groups were age-matched, maturational factors related to craniofacial growth may still influence sphenoid sinus development and volumetric measurements. Since the present study was designed as a cross-sectional morphologic and volumetric comparison rather than a growth-stage analysis, this issue should be addressed in future longitudinal investigations incorporating dedicated skeletal maturation assessment.

Clinical Implications

From a clinical perspective, the present findings do not support the use of sella turcica or sphenoid sinus morphology as reliable screening or predictive markers for unilateral palatal canine impaction. Although these structures are anatomically and developmentally related to the cranial base, the largely non-significant differences observed in this study indicate that their isolated morphologic or volumetric assessment offers limited diagnostic utility in this context. CBCT remains highly valuable when clinically indicated for orthodontic or surgical planning, particularly for the localization of impacted teeth and evaluation of surrounding anatomical structures. However, the present data do not justify the use of sellar or sphenoid morphology alone as a predictive tool for canine impaction. Future studies integrating radiologic findings with other dental, skeletal, or genetic markers may provide a more clinically meaningful risk assessment model.

## 5. Conclusions

This CBCT-based study found that morphological, linear, and volumetric characteristics of the sella turcica and sphenoid sinus were largely comparable between individuals with unilateral palatally impacted maxillary canines and controls. Furthermore, no significant association was observed between the volumes of these two anatomically adjacent structures. These findings suggest that variations in sellar and sphenoid sinus morphology may not represent reliable indicators for unilateral palatal canine impaction. Future studies with larger and multicenter samples are needed to further clarify potential developmental relationships between cranial base structures and dental eruption disturbances.

## Figures and Tables

**Table 1:** Demographic characteristics of the study groups.

Variable	Control (*n* = 35)	UIC (*n* = 35)	*p*-Value
Age (years, mean ± SD)	28.37 ± 12.64	27.00 ± 13.26	0.341
Sex (male/female, %)	17.1/82.9	17.1/82.9	1.000

**Table 2:** Comparison of linear measurements of the sella turcica between groups with effect size estimates.

Parameters (mm)	Control (Mean ± SD)	UIC (Mean ± SD)	*p*-Value (Mann–Whitney U)	Effect Size (r)
Sella width	13.73 ± 0.76	13.10 ± 1.21	0.006 *	0.33
Sella length	9.17 ± 1.21	9.50 ± 1.68	0.372	0.11
Sella depth	7.92 ± 1.01	7.70 ± 1.27	0.155	0.17
Sella diameter	11.74 ± 1.30	11.94 ± 1.35	0.634	0.06

* significance level *p* < 0.05.

**Table 3:** Distribution of sella turcica morphological types according to Axelsson classification [20].

Sella Turcica Morphology	Control *n* (%)	UIC *n* (%)
Bridging	0 (0.0)	2 (5.7)
Irregular dorsum sellae	4 (11.4)	5 (14.3)
Normal	22 (62.9)	21 (60.0)
Oblique anterior wall	3 (8.6)	3 (8.6)
Pyramidal dorsum sellae	6 (17.1)	4 (11.4)

**Table 4:** Distribution of sphenoid sinus pneumatization types.

Sphenoid Sinus Pneumatization Type	Control *n* (%)	UIC *n* (%)
Conchal	0 (0.0)	1 (2.9)
Presellar	3 (8.6)	4 (11.4)
Sellar	12 (34.3)	5 (14.3)
Postsellar	20 (57.1)	25 (71.4)

**Table 5:** Comparison of sella turcica and sphenoid sinus volumes between the UIC and control groups, with effect size estimates.

Parameters (mm^3^)	Control (Mean ± SD)	UIC (Mean ± SD)	*p*-Value (Mann–Whitney U)	Effect Size (r)
Geometric sella volume [0.5 × (L × W × D)]	500.73 ± 108.30	475.89 ± 111.58	0.257	0.14
Sella turcica volume	534.68 ± 67.82	500.50 ± 84.03	0.083	0.21
Sphenoid sinus volume	9448.58 ± 2978.60	8598.83 ± 3085.75	0.369	0.11

**Table 6:** Correlations between sphenoid sinus volume and geometric and three-dimensional sella turcica volumes.

Group	Variable	Geometric Sella Volume	3D Sella Volume
Control	r	−0.088	0.104
	p	0.614	0.554
UIC	r	−0.234	0.037
	p	0.176	0.833

**Table 7:** Comparison of geometric and three-dimensional measurements of sella turcica volume in the study groups.

Group	Measurement	Mean ± SD (mm^3^)	Min–Max (mm^3^)	Paired *t*-Test	*p*-Value
Control	Geometric sella turcica volume	501.76 ± 109.76	302.81–810.30	−1.87	0.071
	3D sella turcica volume	534.11 ± 68.75	415.50–679.95		
UIC	Geometric sella turcica volume	475.89 ± 111.58	309.81–760.79	−1.70	0.099
	3D sella turcica volume	500.50 ± 84.03	354.60–653.32		

**Table 8:** Intraobserver reliability of the measurements using intraclass correlation coefficients.

Variable	ICC	95% CI	*p*-Value
Sella width	0.992	0.983–0.994	<0.0001
Sella depth	0.967	0.943–0.986	<0.0001
Sella diameter	0.973	0.950–0.983	<0.0001
Sella length	0.973	0.949–0.983	<0.0001
Sphenoid sinus volume	0.998	0.995–0.999	<0.0001
3D Sella turcica volume	0.819	0.666–0.894	<0.0001

## Data Availability

The raw data supporting the conclusions of this article will be made available by the authors on request.

## Abbreviations

The following abbreviations are used in this manuscript:

ST	Sella Turcica
CBCT	Cone-Beam Computed Tomography
UIC	Unilateral Impacted Canine
FOV	Field of View
DICOM	Digital Imaging and Communications in Medicine
ICC	Intraclass Correlation Coefficient
mm	millimeter
mm^3^	cubic millimeter
kVp	kilovolt peak
mA	milliampere
